# The prognostic significance of tumor-associated neutrophils and circulating neutrophils in glioblastoma (WHO CNS5 classification)

**DOI:** 10.1186/s12885-022-10492-9

**Published:** 2023-01-06

**Authors:** Xuezhen Wang, Xiaoxia Li, Yufan Wu, Jinsheng Hong, Mingwei Zhang

**Affiliations:** 1 Department of Radiotherapy, Cancer Center, The First Affiliated Hospital of Fujian Medical University, Fuzhou, China; 2 Department of Radiotherapy, National Regional Medical Center, Binhai Campus of the First Affiliated Hospital of Fujian Medical University, Fuzhou, China; 3 Key Laboratory of Radiation Biology of Fujian Higher Education Institutions, The First Affiliated Hospital, Fujian Medical University, Fuzhou, China

**Keywords:** Neutrophils, Glioblastoma, IDH-wildtype, Prognosis, Sensitivity analysis

## Abstract

**Background:**

Tumor-associated neutrophils (TANs) in the tumor microenvironment are prognostic biomarkers in many malignancies. However, it is unclear whether TANs can serve as a prognostic marker for clinical outcomes in patients with glioblastoma (GBM), as classified according to World Health Organization Classification of Tumors of the Central Nervous System, fifth edition (CNS5). In the present study, we analyzed correlations of TANs and peripheral blood neutrophils prior to radiotherapy with overall survival (OS) in GBM (CNS5).

**Methods:**

RNA-seq expression profiles of patients with newly diagnosed GBM (CNS5) were extracted from The Cancer Genome Atlas (TCGA), and The Chinese Glioma Genome Atlas (CGGA). TAN infiltration was inferred using CIBERSORTx algorithm. Neutrophil counts prior to radiotherapy in newly diagnosed GBM (CNS5) were obtained from the First Affiliated Hospital of Fujian Medical University. The prognostic value of TANs and peripheral blood neutrophils before radiotherapy was investigated using Kaplan-Meier analysis and Cox proportional hazards models. The robustness of these findings was evaluated by sensitivity analysis, and E values were calculated.

**Results:**

A total of 146 and 173 individuals with GBM (CNS5) were identified from the TCGA and CGGA cohorts, respectively. High infiltration of TANs was of prognostic of poor OS in TCGA (HR = 1.621, 95% CI: 1.004–2.619) and CGGA (HR = 1.546, 95% CI: 1.029–2.323). Levels of peripheral blood neutrophils before radiotherapy (HR = 2.073, 95% CI: 1.077–3.990) were independently associated with poor prognosis. Sensitivity analysis determined that the E-value of high TANs infiltration was 2.140 and 2.465 in the TCGA and CGGA cohorts.

**Conclusions:**

TANs and peripheral blood neutrophil levels before radiotherapy are prognostic of poor outcomes in GBM (CNS5).

**Supplementary Information:**

The online version contains supplementary material available at 10.1186/s12885-022-10492-9.

## Introduction

Glioblastoma is a highly malignant type of glioma, and accounts for over 50% of emerging glioma cases [1]. Treatment with postoperative chemoradiotherapy is currently the standard of care for glioblastoma. Even though some patients may benefit from the Stupp regimen [2], the overall prognosis remains poor [3], with less than 10% survivorship at 5 years [4]. At present, prognostic predictions for patients with glioblastoma are mainly made based on patient age, Karnofsky Performance Status (KPS), prior treatment, resection range, methylation of O6-methylguanine-DNA methyltransferase (*MGMT*) promoter methylation status, isocitrate dehydrogenase genes (IDH), telomerase reverse transcriptase (*TERT*), and other molecular markers, such as alpha thalassemia/mental retardation syndrome X-linked (ATRX) gene [5, 6]. It is of paramount significance to make overall assessment of patients, and to evaluate potential high-risk factors that affect patient prognosis, to improve the timely adjustment of treatment methods and the accuracy of prognostic assessments.

The World Health Organization Classification of Tumors of the Central Nervous System, fifth edition (WHO CNS5 classification; CNS5) states that an IDH-wildtype diffuse astrocytic glioma in an adult with microvascular proliferation or necrosis or *EGFR* gene amplification or + 7/− 10 chromosome copy number changes or *TERT* promoter mutation should be diagnosed as GBM (CNS5), even if the histological grade was considered low [1]. There are different driver genes, molecular characteristics, and clinical prognosis associated with either IDH mutant or IDH-wildtype glioblastoma [7]. GBM (CNS5) is considered as an independent genotyping for diagnosis based on the fifth edition of the 2021 World Health Organization classification of tumors of the central nervous system [1], thus further advancing the role of molecular neuropathology in CNS tumor classification. Compared with the IDH mutant type, IDH wild type glioblastoma (IDHwt GBM, CNS4) exhibits higher invasiveness, has a poor prognosis, with a median patient survival time ranging from 6 to 15 months [8]. As studies have shown, 30 to 50% of IDHwt GBM (CNS4) demonstrates methylation of the *MGMT* promoter, which is associated with favorable clinical responses to TMZ, and is considered to be a poor prognostic factor [9]. However, the utility of this biomarker may be limited by acquired drug resistance, and disease prognosis still varies greatly. The prognostic value of methylation of *MGMT* promoter [10], the *TERT* promoter, and *EGFR* [11] in GBM (CNS5) remains controversial. It also remains unclear whether heritable factors can contribute to risk stratification for patients [12], and there are likely other factors remaining to be identified that can stratify prognosis for patients with GBM (CNS5). Therefore, there still must be additional reliable biomarkers developed for patient stratification and disease prognosis of patients with GBM (CNS5).

During the progression of glioblastoma, factors such as the tumor microenvironment, and infiltration of non-tumor cells and immune cells influences the gene expression and transcription types of glioblastoma [13], and can result in the interconversion of molecular subtypes. Neutrophils are important members of the tumor microenvironment. Neutrophils exhibit tumor-promoting activity by inducing angiogenesis [14–17], inhibiting T cell activation (immunosuppression) [18–22], inducing genetic instability [23–25], and maintaining tumor cell proliferation [26–29]. Tumor-associated neutrophils (TANs) are also prognostic markers for patients with tumors [30–32], and are closely related to the prognosis of gastric carcinoma [30], breast cancer [33], cholangiocarcinoma [34] and urothelial carcinoma [32]. However, there are few studies on TANs in patients with glioma diagnosed by WHO CNS5 classification, especially for patients with GBM (CNS5), of which the prognostic value is currently unclear. What’s more, at present, studies on hematologic markers of glioma mostly center on preoperative peripheral blood samples [35], and are often disturbed by many factors such as preoperative stress and postoperative infection, which can greatly limit the representation of the real postoperative condition of glioma. Correlative research on the influence of peripheral blood neutrophils on the overall survival of patients with glioma before postoperative radiotherapy has been reported less now, and its influence on the prognosis of glioma is of certain research value.

In the present study, RNA-sequencing (RNA-seq) expression profiles and clinical data from the TCGA database were used to measure the abundance of TANs in the tumor microenvironment by the CIBERSORTx algorithm, and to evaluate the relationship between TANs and clinical prognosis. Moreover, Gene Set Variation Analysis (GSVA) enrichment analysis was performed to explore differences in biological characteristics between the high and low TANs groups, and the CGGA database was used for external verification. In addition, a retrospective analysis was made on the levels of peripheral blood neutrophils before radiotherapy for patients with GBM (CNS5), and the prognostic significance of this metric was determined for GBM (CNS5).

## Methods

### Data collection

#### TCGA database

Level 3 gene expression profiles (level 3 data) for glioblastoma patients were downloaded from TCGA (The Cancer Genome Atlas) database (https://portal.gdc.cancer.gov/). Clinical data such as sex, age, and overall survival (OS) were also downloaded from TCGA data portal. The molecular pathological data regarding IDH, *MGMT* promoter methylation, *TERT* promoter mutation, and + 7/− 10 chromosome copy number was extracted from a published database [36].The detailed inclusion criteria included: 1) primary glioblastoma; 2) according to WHO CNS5 classification, an IDH-wildtype diffuse astrocytic glioma in adults with microvascular proliferation or necrosis, or 1 or more of 3 genetic parameters [*EGFR* gene amplification, combined gain of entire chromosome 7 and loss of entire chromosome 10 (+ 7/− 10), *TERT* promoter mutation] should be diagnosed as GBM (CNS5) [1]. For further categorization of GBM, GBM with histologic diagnosis (histoGBM, CNS5) was defined as IDH-wildtype diffuse astrocytoma with microvascular proliferation or necrosis, and molecular diagnostic GBM (molGBM, CNS5) was defined as IDH-wildtype diffuse astrocytoma that did not have the histologic appearance described above, if any one or a combination of *EGFR* gene amplification, + 7/− 10, or *TERT* promoter mutation were present [37, 38]. Exclusion criteria included: 1) recurrent glioblastoma; 2) incomplete records in grade or IDH mutation status; 3) patients with missing survival data or OS < 90 days, or without definitive OS.

#### CGGA database

The CGGA RNA sequencing (RNA-seq) dataset (mRNAseq_693, mRNAseq_325) and corresponding molecular and clinical information were acquired from the Chinese glioma genome atlas (CGGA) database (http://www.cgga.org.cn/index.jsp), which provides information such as age, sex, grade, subtype, *MGMT* promoter status, IDH status, and follow-up data of each patient. Inclusion and exclusion criteria were consistent with those for the TCGA dataset.

### Acquisition of tumor-associated neutrophil data

By using TCGA and CGGA RNA-seq data, the content of GBM (CNS5) TANs was computed by CIBERSORTx, an analysis tool (https://cibersortx.stanford.edu/) [39]. The content of TANs was considered a continuous variable, and a binary variable was obtained by establishing a cut-off point (cut) by using “survMisc” package (https://cran.r-project.org/web/packages/survMisc/index.html) [40], where TANs content below or equal to the cut-off point was considered as the low group, and the high group was patients whose TANs content was higher than the cut-off point.

### Biological enrichment analysis

GSVA is a nonparametric and unsupervised approach, that is used to estimate changes in pathways and biological activity in a sample of an expression dataset. The gene sets “c2.cp.kegg.v7.5.1.symbols.gmt” and “h.all.v7.5.1.symbols.gmt”, which were obtained from the MSigDB database (http://www.gsea-msigdb.org/gsea/login.jsp), were used for performing GSVA analysis. GSVA was carried out using the R “GSVA” package to evaluate the enrichment score of the pathways in the high-TANs and low-TANs groups [41]. The correlation between the enrichment score and the level of TANs was evaluated by Spearman correlation analysis. We also evaluated correlations between functional molecules involved in the tumor-promoting mechanism of neutrophils in the tumor microenvironment, including *CXCR4, TGFBR1, CXCR1, CD86, PILRA, LILRB2, CD200R1, TNFSF10, S100A9, S100A8, PROK2, MMP9, AGTR1, IFNAR1, IFNB1, PDGFB,* and *ARG1*. Furthermore, we investigated the correlation between TANs infiltration and the expression of neutrophil functional molecules and apoptotic genes.

### Prognostic value of peripheral blood neutrophils in a radiation cohort

#### Ethical approval of the study protocol

The study protocol was approved by the Ethics Review Board for Human Research of The First Affiliated Hospital of Fujian Medical University (Fujian, China), (approval No. [2015]084–1), and all participants gave written informed consent.

#### Research design

A retrospective cohort study was adopted to collect data from all patients with GBM (CNS5) treated in the radiotherapy department from September 2013 to June 2020. Pathological diagnoses were reevaluated and confirmed by two different pathologists from the Pathology Department of The First Affiliated Hospital of Fujian Medical University. Inclusion criteria: 1) GBM (CNS5); 2) surgery and post-operative intensity modulated radiation therapy (IMRT) were performed; 3) the hematological examination data was completed within 1 week prior to radiotherapy; 4) patients with complete follow-up information. Exclusion criteria: 1) antitumor therapy was performed before surgery (including radiotherapy, chemotherapy, biotherapy, immunotherapy, or targeted therapy); 2) patient suffered from an infectious diseases such as septicemia during hematological examination; 3) the presence of two or more tumors simultaneously; 4) complications with hematological diseases; 5) complications with immune system diseases; 6) transfusion history within 1 month; 7) history of long-term glucocorticoid treatment.

#### Demographic and clinicopathologic variables and outcomes

Demographic and clinicopathologic variables included sex, age and methylation status of the *MGMT* promoter. The level of neutrophil counts in routine blood parameters within 1 week prior to radiotherapy were also reported. A cut-off point was obtained using the “survMisc” package (https://cran.r-project.org/web/packages/survMisc/index.html), and the level of neutrophil counts were divided into a high group and a low group. Follow-up, including further consultation and / or telephone follow-up, ended in December 2020.

### Statistical analysis

R version 4.0.1 (R Foundation for Statistical Computing, Vienna, Austria;www.r-project.org) was used for statistical analysis. The categorical variables were presented as number and percentage (N, %), and Pearson’s Chi-Square test was used for comparison between groups. The correlation between the content of functional molecules and TANs, as mentioned above, was confirmed by Spearman correlation analysis. A correlation coefficient greater than 0.3 was defined as a significant correlation [42]. The overall survival (OS) was estimated from the date of diagnosis to death or the last follow-up, which was calculated by Kaplan-Meier method and the log-rank test. The univariate and multivariate Cox regression models were performed to determine potential prognostic factors. Sensitivity analysis: as to the TANs computed by CIBERSORTx, the results of multivariate Cox regression analysis of TANs infiltration were repeatedly validated, to verify the robustness of the determination of independent risk factors for high and low, identified by different in silico algorithms. Adopt E-value [43] was used to evaluate the extent to which unmeasured confounding factors influenced the results All statistical tests were two-sided, and a *p*-value of *P* < 0.05 was considered significant.

## Results

### Demographic and clinicopathologic characteristics

**Fig. 1:**
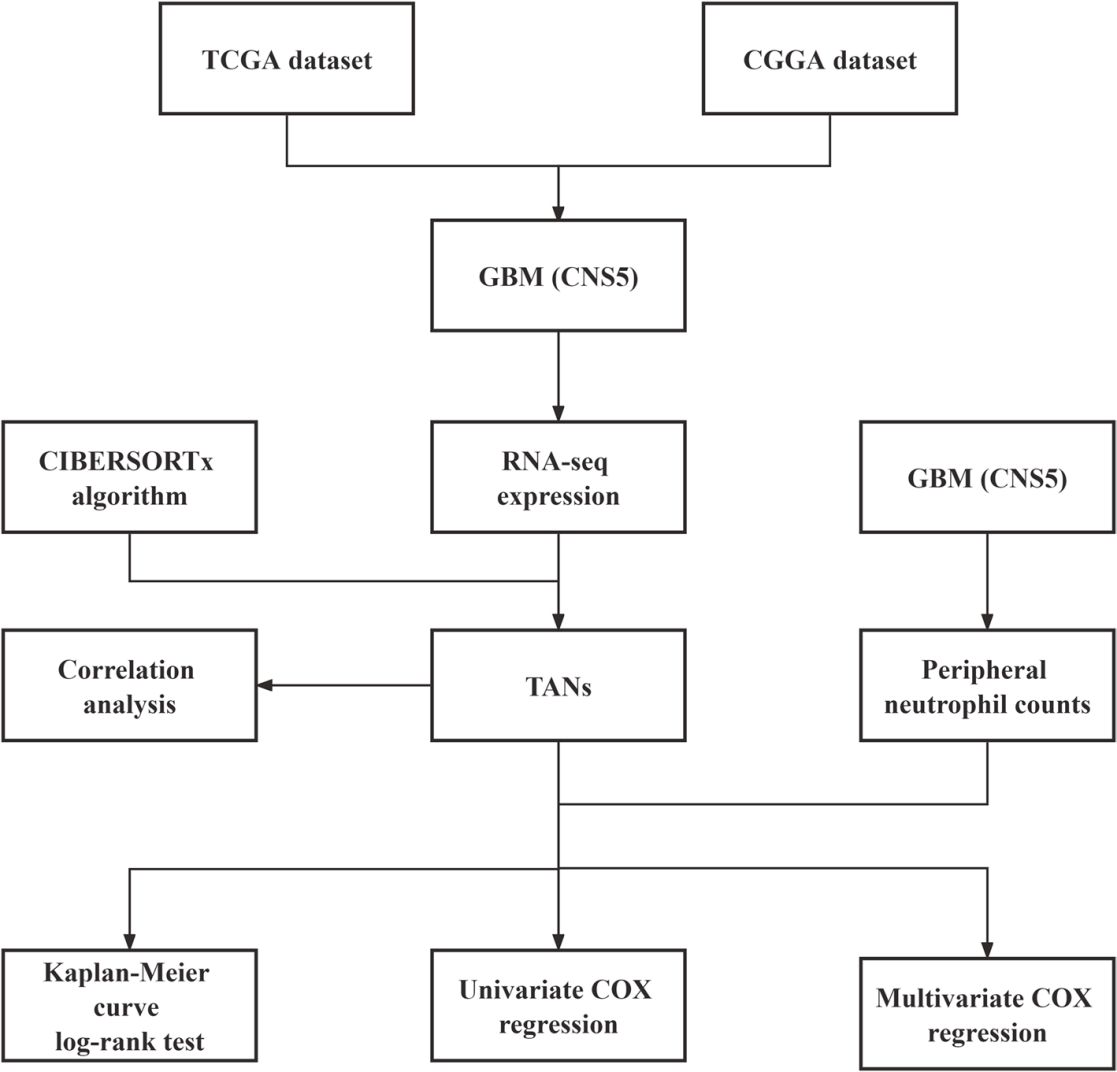
Flowchart of sample data analysis

The study design is shown in Fig. 1. A total of 146 eligible GBM (CNS5) patients were identified in the TCGA database and selected in this study. In the TCGA dataset, there was no statistically significant difference in age, sex, whether radiation or chemotherapy was performed, and *MGMT* promoter methylation status among patients in the TANs high group and low group (*P* > 0.05). Similarly, the CGGA RNA-seq database with 173 GBM (CNS5) samples was used as a validation cohort. In CCGA dataset, there were no statistically significant differences in age, sex, whether radiation was performed, and *MGMT* promoter methylation status (*P* > 0.05) (Table 1). The distribution of the TANs levels between molGBM (CNS5) and histoGBM (CNS5) are shown in Supplementary file 1.

**Table 1:** Summary of clinicopathological features of glioblastoma (CNS5) patients in the TCGA and CGGA cohorts

	TCGA				CGGA			
Variables	Total (*n*=146)	High group (*n*=100)	Low group (*n*=46)	*p*	Total (*n*=173)	High group (*n*=38)	Low group (*n*=135)	*p*
Age				0.606				0.325
<60	70 (48%)	46 (46%)	24 (52%)		114 (66%)	22 (58%)	92 (68%)	
≥60	76 (52%)	54 (54%)	22 (48%)		59 (34%)	16 (42%)	43 (32%)	
Sex				0.236				0.589
Female	53 (36)	40 (40%)	13 (28%)		68 (39%)	13 (34%)	55 (41%)	
Male	93 (64)	60 (60%)	33 (72%)		105 (61%)	25 (66%)	80 (59%)	
Radiation				0.918				0.298
NO	20 (14%)	13 (13%)	7 (15%)		25 (14%)	3 (8%)	22 (16%)	
YES	126 (86%)	87 (87%)	39 (85%)		148 (86%)	35 (92%)	113 (84%)	
Chemotherapy				0.524				0.016
NO	35 (24%)	26 (26%)	9 (20%)		37 (21%)	14 (37%)	23 (17%)	
YES	111 (76%)	74 (74%)	37 (80%)		136 (79%)	24 (63%)	112 (83%)	
MGMT promoter				0.593				0.248
Methylated	51 (35%)	33 (33%)	18 (39%)		71 (41%)	12 (32%)	59 (44%)	
Un-methylated/Unknown	95 (65%)	67 (67%)	28 (61%)		102 (59%)	26 (68%)	76 (56%)	
TERT promoter				0.008				-
Mutant	41 (28%)	21 (21%)	20 (43%)		-	-	-	
Unknown	103 (71%)	78 (78%)	25 (54%)		-	-	-	
WT	2 (1%)	1 (1%)	1 (2%)		-	-	-	
KPS				0.025				-
<70	25 (17%)	22 (22%)	3 (7%)		-	-	-	
≥70	88 (60%)	60 (60%)	28 (61%)		-	-	-	
Unknown	33 (23%)	18 (18%)	15 (33%)		-	-	-	
Group				<0.001				-
histoGBM	113 (77%)	89 (89%)	24 (52%)		-	-	-	
molGBM	33 (23%)	11 (11%)	22 (48%)		-	-	-	

### Survival of patients and potential prognostic factors for OS

#### TCGA dataset

**Fig. 2:**
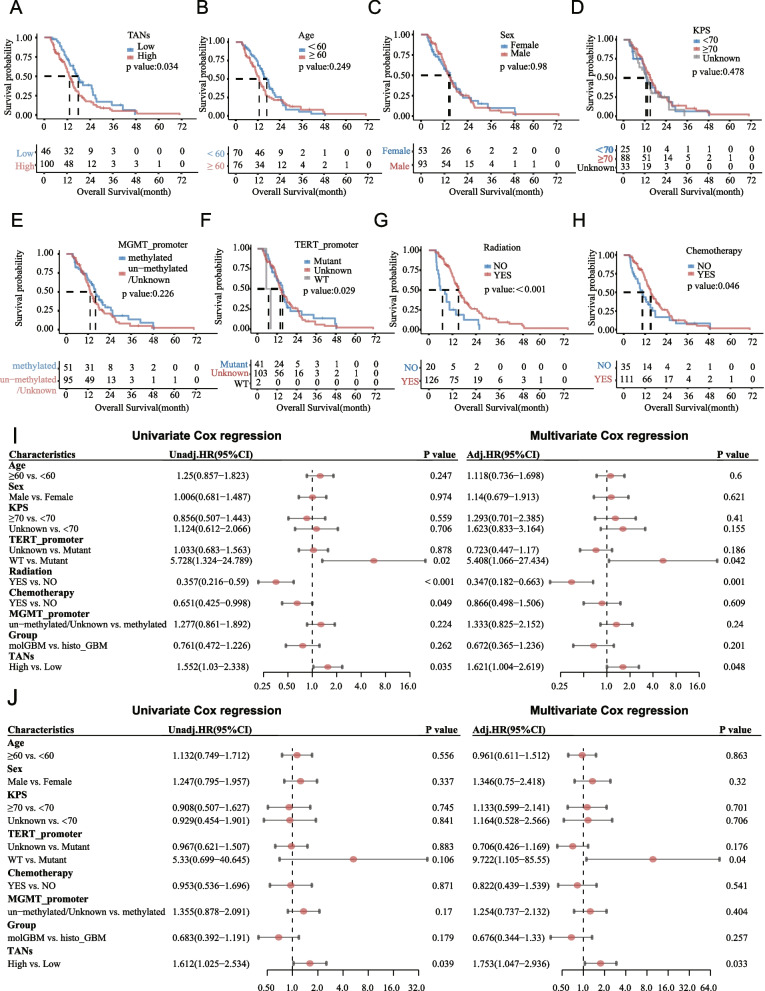
KM survival curves of patients based on TANs levels (**A**), age (**B**), sex (**C**), KPS (**D**), MGMT promoter status (**E**), TERT promoter status (**F**), radiation status (**G**), chemotherapy status (**H**). Univariate and multivariate Cox analysis of TANs level and patient survival in the entire GBM (CNS5) cohort in the TCGA dataset (**I**). Univariate and multivariate Cox analysis of TANs level and patient survival in patients treated with radiation in the TCGA dataset (**J**)

In the TCGA dataset, clinical follow-up was available for 146 patients and KM survival curve for OS was performed (Fig. 2A-H). The median survival time of patients in the TANs high group was 13.2 months, and was 17.7 months for patients in the TANs low group, and there was a statistically significant difference in overall survival between the groups (*P* = 0.034; Fig. 2A). Of note, age, sex, KPS and *MGMT* promoter methylation status did not significantly affect OS (*P* = 0.249, 0.98, 0.478, and 0.226, respectively; Fig. 2B-E). Meanwhile, patients who received radiation or chemotherapy had longer OS (*P* < 0.001 and *P* = 0.046, respectively; Fig. 2G, H). In the TCGA dataset, univariate Cox analysis have shown that the infiltration of TANs (HR = 1.552, 95% CI: 1.03–2.338), radiation (HR = 0.357, 95%CI: 0.216–0.59), and chemotherapy (HR = 0.651, 95% CI: 0.425–0.998) were factors that significantly influenced the prognosis of patients with GBM (CNS5) (Fig. 2I). Multivariate Cox regression showed that the infiltration of TANs (HR = 1.621, 95% CI: 1.004–2.619) and radiation (HR = 0.347, 95% CI: 0.182–0.663) were independent factors influencing the prognosis of patients with GBM (CNS5) (Fig. 2I). The subgroup analysis of 126 patients who received radiotherapy confirmed that high TANs infiltration was associated with shorter OS (HR (95%CI) = 1.753 (1.047–2.936)) (Fig. 2J).

#### External validation

In the CGGA dataset, follow-up details were available for 173 patients. The median survival time of patients in the TANs high group was 12.6 months, and was 15.8 months for patients in the TANs low group; there were statistically significant differences in overall survival between the two groups (*P* = 0.002; Fig. S1A). Of note, patients less than 60 years of age or who received chemotherapy had longer OS (*P* = 0.016 and *P* < 0.001, respectively; Fig. S1B, F) In the CGGA dataset, univariate Cox analysis revealed that the infiltration of TANs (HR = 1.799, 95% CI: 1.227–2.637), age (HR = 1.5, 95% CI: 1.076–2.091) and chemotherapy (HR = 0. 419, 95% CI: 0.285–0.616) were factors influencing the prognosis of patients with GBM (CNS5) (Fig. S1G). Multivariate Cox regression showed that the level of TANs infiltration (HR = 1.546, 95% CI: 1.029–2.323), age (HR = 1.461, 95% CI: (1.041–2.052) and chemotherapy (HR = 0.414, 95% CI: 0.268–0.64) were independent prognostic factors for OS of GBM (CNS5) patients.

### Sensitivity analysis

**Fig. 3:**
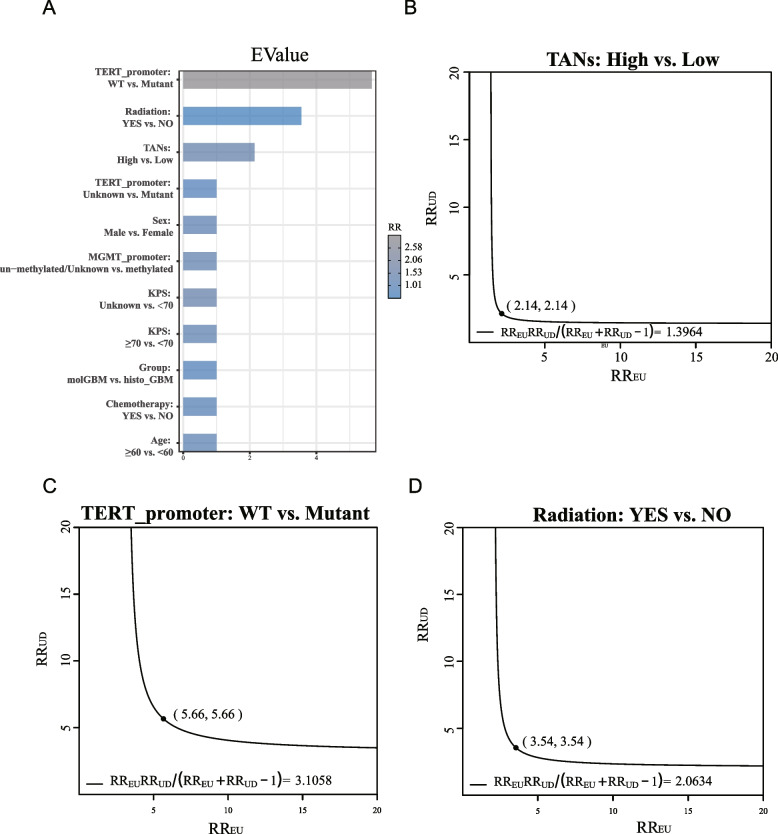
Sensitivity analyses in the TCGA cohort

In the TCGA dataset, after adjusting for patient age, sex, radiation, chemotherapy, and methylation of *MGMT* promoter, the RR = 1.396 and E-value (95%CI) = 2.140(1.055–3.281) were determined for death in the TANs high group (Fig. 3A, B). The RR and E-value of *TERT* promoter and radiation were shown in Fig. 3C and D, respectively. In the CGGA dataset, the RR = 1.546 and E-value (95%CI) = 2.465(1.202–4.076) were determined for death in the TANs high group, and the RR = 1.461 and E-value = 2.28 for the aged ≥60 years group (Fig. S2A, B).

### Biological enrichment analysis

**Fig. 4:**
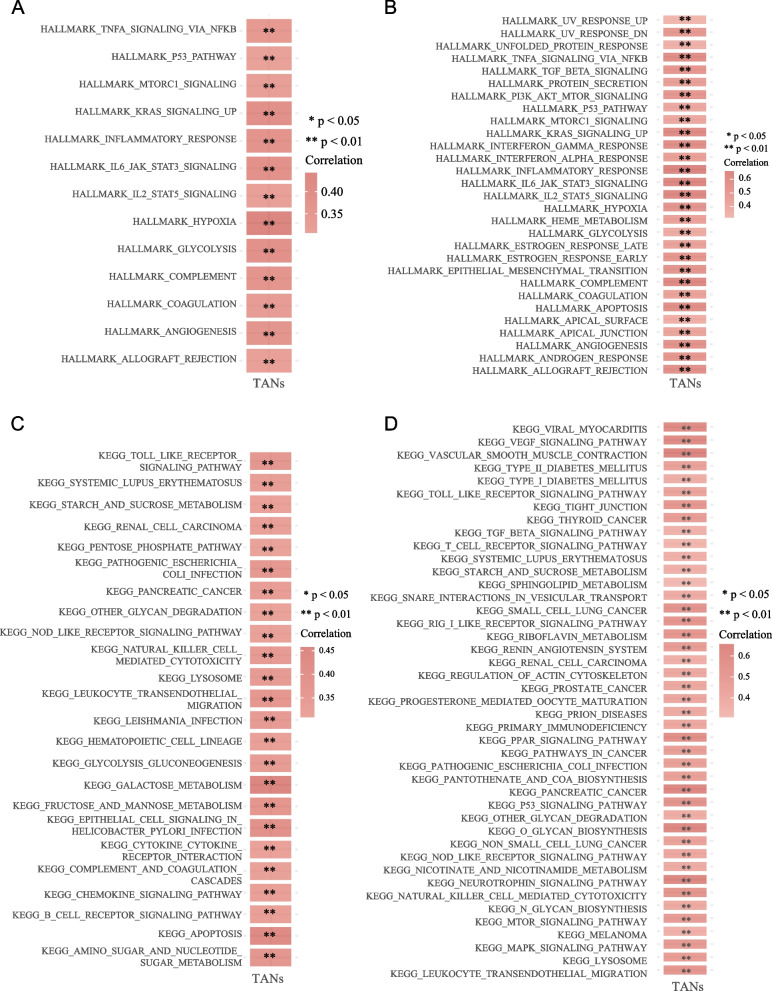
Correlation analysis between KEGG pathways and TANs levels in the TCGA cohort (**A**), and the CGGA cohort (**B**) via GSVA. Correlation analysis of hallmark pathways and TANs levels in the TCGA cohort (**C**), and the CGGA cohort (**D**) via GSVA

**Fig. 5:**
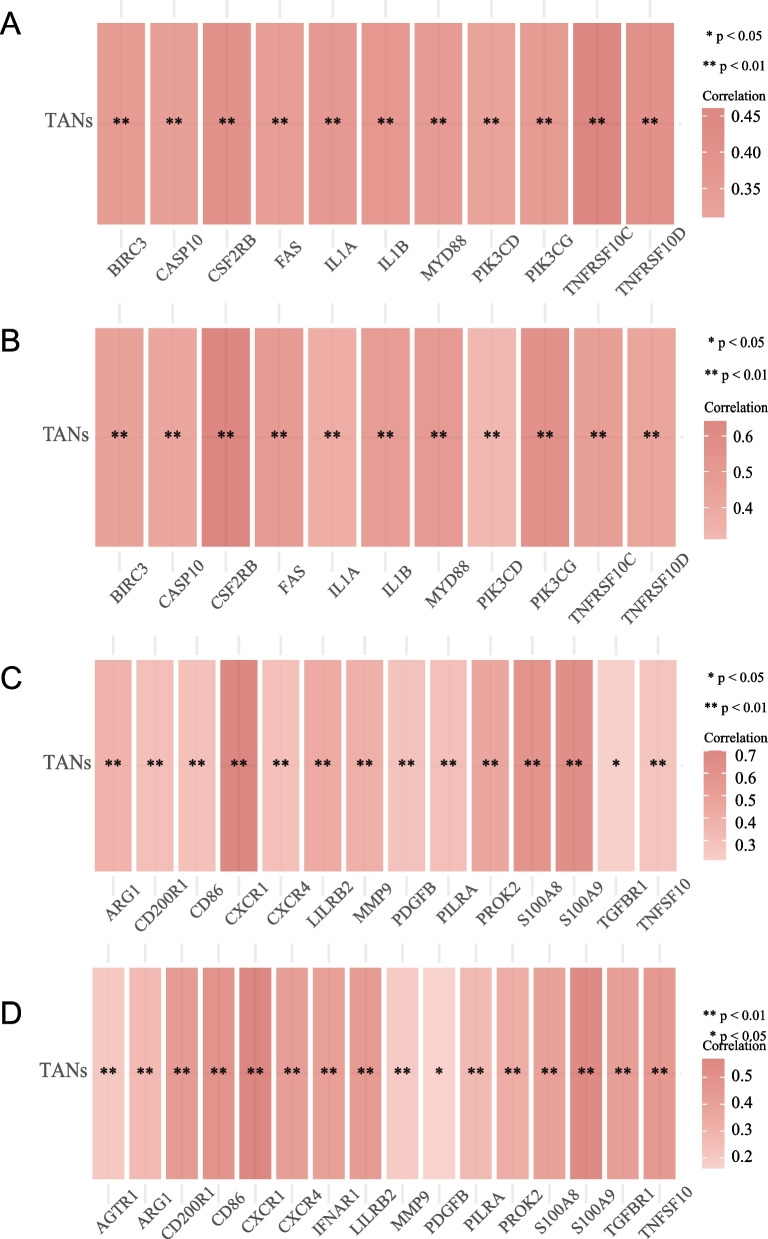
Correlation analysis between TANs levels and expression of apoptosis-related genes in the TCGA cohort (**A**), and the CGGA cohort (**B**). Correlation analysis between TANs levels and expression of neutrophil function-related genes in the TCGA cohort (**C**), and the CGGA cohort (**D**)

Heatmaps were generated indicating Spearman correlation coefficients greater than 0.3 or less than 0.3. Correlation analysis between TANs infiltration level and GSVA enrichment scores showed that TANs levels ware significantly correlated with hypoxia (TCGA cohort: *r*^2^ = 0.441, *P* < 0.001; CGGA cohort: *r*^2^ = 0.538, *P* < 0.001) (Fig. 4A, B, Table S1) and apoptosis (TCGA cohort: *r*^2^ = 0.431, *P* < 0.001; CGGA cohort: *r*^2^ = 0.638, *P* < 0.001) (Fig. 4C, D, Table S2). The level of TANs infiltration was significantly correlated with the expression of the apoptotic genes *TNFRSF10C* (TCGA cohort: *r*^2^ = 0.460, *P* < 0.001; CGGA cohort: *r*^2^ = 0.461, *P* < 0.001) and *TNFRSF10D* (TCGA cohort: *r*^2^ = 0.397, *P* < 0.001; CGGA cohort: *r*^2^ = 0.426, *P* < 0.001) (Fig. 5A, B, Table S3). Additionally, TANs were found to be significantly correlated with the expression of neutrophil function-related genes, including *CXCR1* (TCGA cohort: *r*^2^ = 0.700, *P* < 0.001; CGGA cohort: *r*^2^ = 0.569, *P* < 0.001) and *S100A9* (TCGA cohort: *r*^2^ = 0.628, *P* < 0.001; CGGA cohort: *r*^2^ = 0.542, *P* < 0.001) (Fig. 5C, D, Table S4).

### The prognostic value of peripheral blood neutrophils in a radiation cohort

**Fig. 6:**
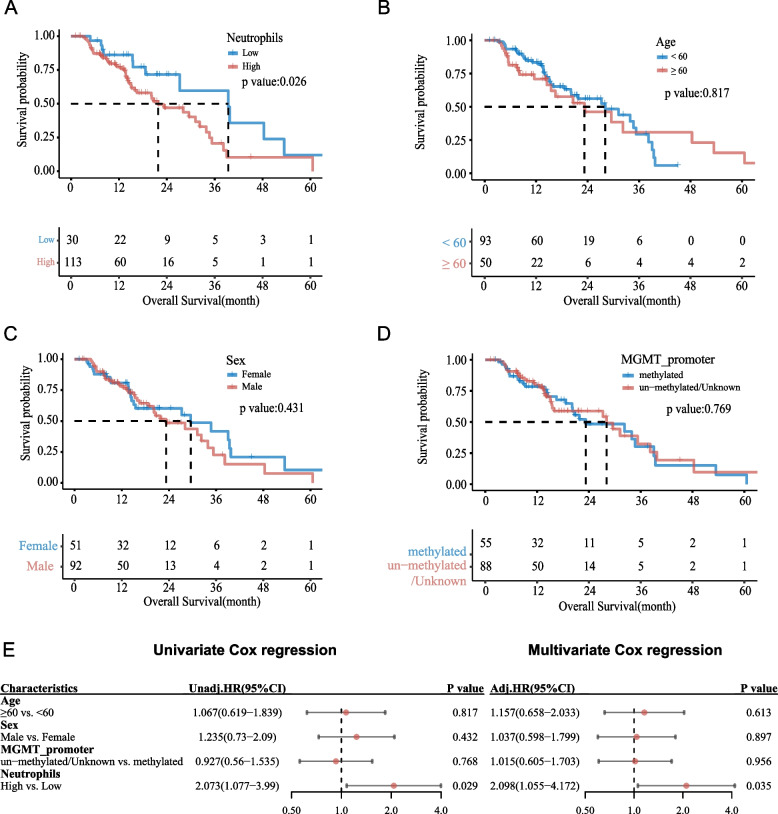
KM survival curves of peripheral blood neutrophils (**A**), age (**B**), sex (**C**), MGMT-promoter status (**D**), and univariate and multivariate Cox analyses (**E**) of peripheral blood neutrophils before radiation in the patient dataset from The First Affiliated Hospital of Fujian Medical University

In the radiation cohort, 143 patients with GBM (CNS5) were included, and there were no statistically significant differences in age, sex, radiation, chemotherapy, or methylation of *MGMT* promoter between the peripheral blood neutrophil high and low groups before radiation (Table S5). The correlation between peripheral blood neutrophils and survival before radiation was analyzed; 50 patients died at the end of follow-up, with a median survival time of 21.8 months in the peripheral blood neutrophil high group, and 13 patients died in the low group, with a median survival time of 39.4 months. The overall survival of patients in the high peripheral blood neutrophil group was significantly shorter than that in low group (*P* = 0.026; Fig. 6A). Kaplan Meier survival curves were generated for patients based on age, sex, and MGMT promoter methylation status (Fig. 6B-D). In accordance with the univariate and multivariate Cox regression models: the level of peripheral blood neutrophils before radiation (Univariate Cox regression: HR = 2.073, 95% CI: 1.077–3.990; Multivariate Cox regression: HR = 2.098, 95% CI: 1.055–4.172) was an independent risk factor affecting the overall survival of patients with GBM (CNS5) (Fig. 6E).

## Discussion

While the integrated WHO CNS5 classification has advantages for guiding clinical diagnosis compared with previous simple histological diagnosis, it also further increases the heterogeneity of GBM cohorts and sets higher requirements for evaluating prognosis. Despite some research efforts in IDHwt GBM (CNS4), it remains unknown as to whether TANs could serve as a prognostic biomarker in patients diagnosed as GBM (CNS5). Patients diagnosed with GBM (CNS5) were included in this study, and we found that high TANs level remains an independent prognostic factor for poor OS of GBM (CNS5) [TCGA cohort: HR (95%CI) = 1.621(1.004–2.619); CGGA cohort: HR (95%CI) = 1.526(1.029–2.323)]. Moreover, the level of TANs infiltration was significantly correlated with the expression of apoptotic genes, including *TNFRSF10C* and *TNFRSF10D*, and with expression of the neutrophil marker genes *CXCR1*, *S100A9*. In order to investigate the effect of peripheral blood neutrophils on the prognosis of GBM (CNS5), data from 143 patients was analyzed. Peripheral blood neutrophils before radiotherapy was an independent prognostic factor for OS [HR (95%CI) = 2.098 (1.055–4.172)]. Neutrophils are present in most solid tumors microenvironments [44–49], and are important non-malignant cells found in the tumor microenvironment [50]. Neutrophil infiltration influences the response to different anticancer therapies, and high neutrophil infiltration is associated with a poor response to radiotherapy [51]. In this study, a subgroup analysis of 126 patients who received radiotherapy confirmed that high TANs infiltration was associated with shorter OS [HR (95%CI) = 1.753 (1.047–2.936)].

Most current studies on the prognostic significance of TANs do not agree on the relevant biomarkers of neutrophils, which may result in a bias in prognostic estimates. By analyzing three groups of operative specimens of patients with gastric cancer who received total or partial gastrectomy independently at two medical centers, Zhang et al. found that high infiltration of TANs in gastric tissue suggests a better prognosis [30]. Zhao et al. [52] demonstrated that high infiltration of TANs in gastric tissue suggests a poor prognosis. Causes for this difference may be that CD66b was used to mark neutrophils in the former study, while CD15 was used to mark neutrophils in the latter study. CD15 can be expressed not only in neutrophils, but also in monocytes, eosinophils, and tumor cells, among other cell types. As a consequence, the RNA-seq data of TCGA and CGGA datasets were analyzed in the present study by CIBERSORTx in an exploratory way, to infer the neutrophil infiltration levels and avoid potential biases introduced by evaluating only specific neutrophil markers.

TANs are involved in malignant transformation and angiogenesis in numerous preclinical and clinical studies [53–57]. Arora et al. demonstrated that higher levels of *S100A8* (median survival: High vs. Low = 12.73 months vs. 15.1 months, respectively; *P* = 0.0009) and *S100A9* (median survival: High vs. Low = 12.67 months vs. 15.03 months, respectively; *P* = 0.0005) gene expression was associated with poor prognosis in GBM (CNS4) patients [58]. By releasing angiogenic factors including S100A8 and S100A9, as well as activating vascular endothelial growth factors A (VEGFA) in the extracellular matrix and MMP9, tumor angiogenesis was maintained by neutrophils [14–17]. This angiogenic effect was also found in hepatocellular carcinoma, gastric cancer, and nasal carcinoma [59–61]. *S100A8/S100A9* co-expression in hepatocellular carcinoma cells promotes malignant progression by induction of ROS, down-regulation of p38 MAPK signaling, cell survival, and resistance to tumor necrosis factor (TNF)-α-induced apoptosis [62]. Li et al. report that high expression of *MMP9* is associated with the pathological grading of gliomas and predicts poor prognosis [OS: HR (95%CI) = 1.171(1.018–1.346), PFS: HR (95%CI) = 1.146(1.012–1.299)]. Patients with lower *MMP9* expression are more likely to benefit from TMZ treatment regardless of *MGMT*-methylation status [63]. Furthermore, neutrophil can stimulate dormant cancer cells through release of MMP9 which can produce epitopes that bind to tumor integrins and trigger the proliferation of cancer cells [26, 27]. It has been reported that CXCR1 mRNA expression is significantly higher in patients with glioma than in normal individuals [64]. The tumor-promoting activity of neutrophils was related to growth factors and chemotactic factors [53, 65, 66], and CXCR was involved in promoting neutrophil maturation, survival, and recruitment [18, 67–69]. TNFRSF10C is a protein that belongs to the TNFRSF family that binds to tumor necrosis factor-related apoptosis-inducing ligand (TRAIL) and inhibits intracellular apoptotic signaling pathways [70]. *TNFRSF10D* expression is associated with prostate cancer and TNFRSF10D is a direct effector *p53* and ERK signaling pathways [71]. Although the prognostic value of TNFSF10C and TNFRSF10D has not been previously investigated in glioma, these proteins have the potential to be used as novel biomarkers.

Neutrophils are classical congenital immune cells that are important members of the tumor immune micro-environment. Neutrophils in peripheral blood and tissues are of the same origin [72, 73]. A clinical study of 1233 patients undergoing radical radiotherapy demonstrated a significant association between elevated blood neutrophil counts and reduced 3-year OS [74]. In view of current glioma studies, the clinical studies to explore the prognostic value of neutrophils have mostly focused on preoperative peripheral blood samples, and most of evaluated the ratio of neutrophils to lymphocytes [35], which may not truly reflect the prognostic value of peripheral blood neutrophils, given that this index is susceptible to lymphocyte interference. K. Takakura et al. [75] demonstrated that NLR was significantly associated with high density CD20+ lymphocytes (*P* = 0.031) and CD163+ macrophagocytes (*P* = 0.023), but not with CD66b + neutrophils (*P* = 0.397). Also, the correlation between neutrophils and prognosis may also be influenced by the location in tumors. Immunohistochemical studies on operative specimens of esophageal squamous carcinoma found that 5-year rates of DFS and OS were 20 and 26.7%, respectively, in patients with increased CD66+ intratumoral neutrophils, but 51.1 and 55.5%, respectively, in patients with decreased CD66+ neutrophils, suggesting that CD66+ neutrophils are an independent prognostic factor of DFS (HR = 2.174 (1.249–3.784), *P* = 0.006) and OS (HR = 1.858 (1.038–3.325), *p* = 0.037). No prognostic significance of peritumoral neutrophils was noted [76]. The correlation between neutrophils and prognosis was also influenced by the time of specimen collection, especially peripheral blood specimens. Whereas most patients with glioma are treated with surgery, there may be differences in tumor burden status after operation compared with pre-operation. Meanwhile, neutrophil infiltration was shown to associate with radiotherapy sensitivity [77]. Presently, there are few reports on the association with postoperative peripheral blood neutrophil before radiotherapy and OS of GBM (CNS5). Therefore, the time point before radiotherapy used in this study, with strict inclusion and exclusion criteria to avoid the influences brought by postoperative surgical stress or postoperative infection, may ensure better evaluation of the effects of the overall immune status of patients with glioma before radiotherapy. Our results showed that the level of peripheral blood neutrophils before radiotherapy was an independent risk factor that affects the prognosis of patients with GBM (CNS5) suggesting that immune status before radiotherapy affects the survival of patients with glioma.

There are several limitations of this study that need to be discussed. First, this study is an observational study, and it is unknown to what extent unmeasured confounders may have influenced the results. In order to reduce the interference of confounding factors, the study used multivariate analysis to adjust as many confounding factors as possible. Additionally, E-values were calculated to assess the impact of unmeasured confounders. However, confounding factors such as the precise types, dose, course and comedication of chemotherapy and radiotherapy were not fully documented in the database, and were therefore unable to be evaluated in this study. Secondly, levels of TANs evaluated by CIBERSORTx is calculated by mRNA-seq, which lacks data validation on a cell-by-cell level. Furthermore, the interactions between blood neutrophils and TANs and the tumor-promoting or tumor-inhibiting mechanisms of neutrophils were not been explored in depth. The results of this study need to be validated by prospective multi-center randomized trials with a larger patient population in the future.

## Conclusions

TANs can be used as a prognostic marker for patients with GBM (CNS5). Patients whose tumors have a high infiltration of TANs have a worse prognosis.

## Supplementary Information

**Additional file 1: Fig. S1.** KM survival curves of patients based on TANs levels **(A)**, age **(B)**, sex **(C)**, MGMT promoter status **(D)**, radiation status **(E)**, chemotherapy status **(F).** The univariate and multivariate Cox analyses of TANs levels and patient survival in the whole-cohort GBM(CNS5) patients in dataset of CGGA **(G)**.

**Additional file 2: Fig. S2.** Sensitivity analyses in the CGGA cohort.

**Additional file 3: Table S1.** Correlation analysis of TANs levels with GSVA scores of hallmark gene sets in dataset of TCGA and CGGA, respectively.

**Additional file 4: Table S2.** Correlation analysis of TANs levels with GSVA scores of KEGG pathways in dataset of TCGA and CGGA, respectively.

**Additional file 5: Table S3.** Correlation analysis of TANs levels with apoptotic-related genes in dataset of TCGA and CGGA, respectively.

**Additional file 6: Table S4.** Correlation analysis of TANs levels with neutrophils function-related marker genes in dataset of TCGA and CGGA, respectively.

**Additional file 7: Table S5.** Characteristics of the study population based on the level of peripheral blood neutrophils before radiotherapy.

**Additional file 8: Supplementary File 1.** *The* distribution *of the* TANs levels between molGBM and histoGBM.

## Data Availability

Data are available from the corresponding authors on reasonable request.
