# Supplementary material for: The prognostic significance of tumor-associated neutrophils and circulating neutrophils in glioblastoma (WHO CNS5 classification)
Source: BMC Cancer. 2023 Jan 6;23:20. doi: 10.1186/s12885-022-10492-9 (PMC9817270; doi:10.1186/s12885-022-10492-9)

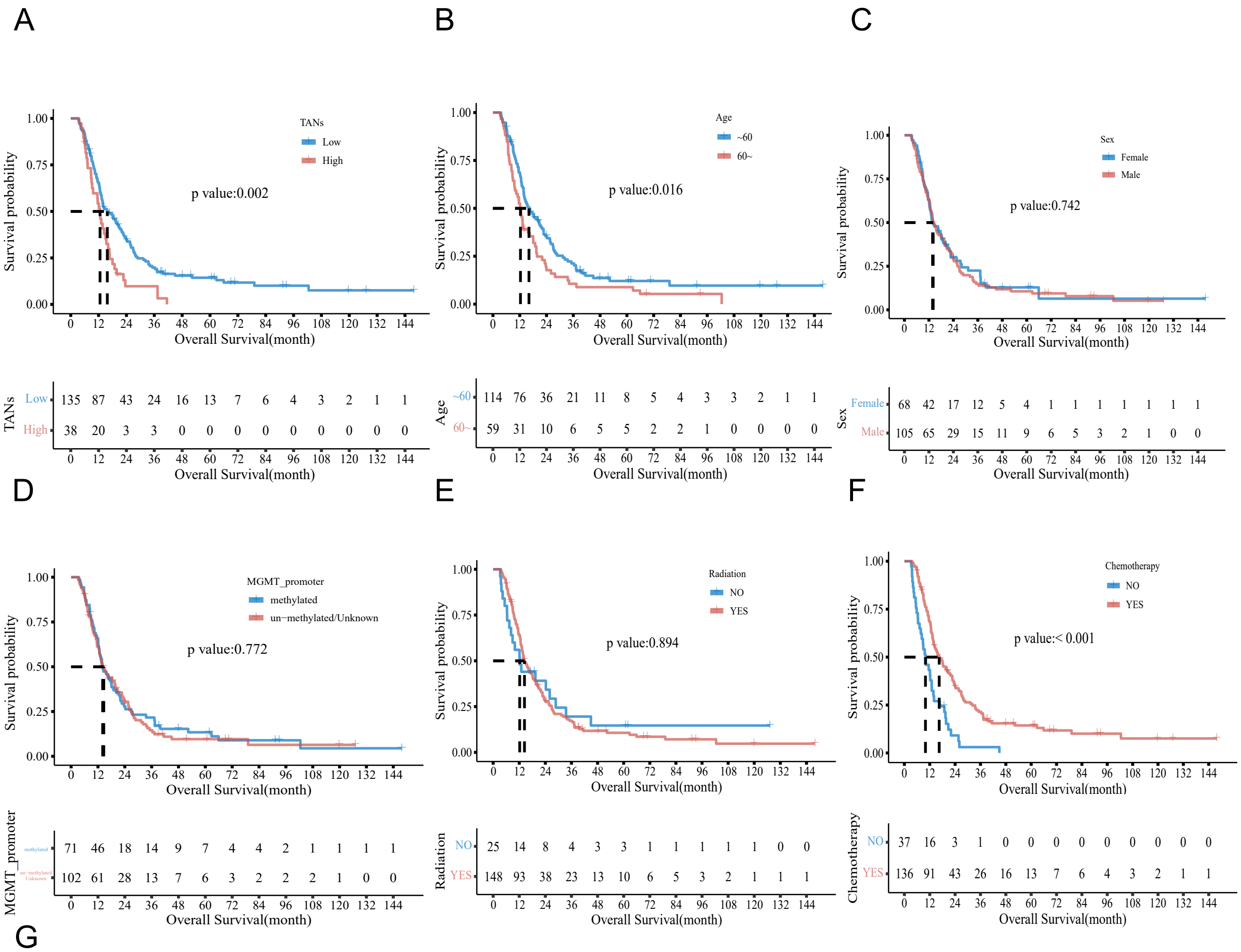

G

Charateristics	Unadj.HR(95%CI)	P value	Adj.HR(95%CI)	P value
**Age**				
60~ vs. ~60	1.5(1.076−2.091)	0.017	1.461(1.041−2.052)	0.028
**Sex**				
Male vs. Female	1.056(0.758−1.472)	0.746	0.984(0.687−1.41)	0.932
**Radiation**				
YES vs. NO	1.033(0.644−1.655)	0.893	1.282(0.752−2.186)	0.362
**Chemotherapy**				
YES vs. NO	0.419(0.285−0.616)	< 0.001	0.414(0.268−0.64)	< 0.001
**MGMT_promoter**				
un−methylated/Unknown vs. methylated	1.049(0.757−1.453)	0.774	1.067(0.746−1.526)	0.724
**TANs**				
High vs. Low	1.799(1.227−2.637)	0.003	1.546(1.029−2.323)	0.036

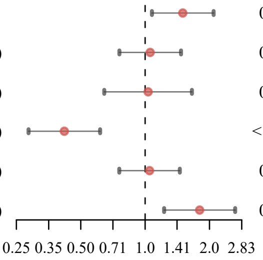

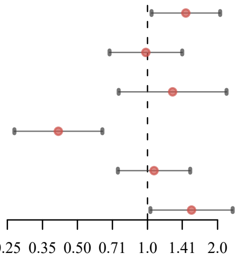

Supplement: Supplementary file 1 — Additional file 1: Fig. S1. KM survival curves of patients based on TANs levels (A), age (B), sex (C), MGMT promoter status (D), radiation status (E), chemotherapy status (F). The univariate and multivariate Cox analyses of TANs levels and patient survival in the whole-cohort GBM(CNS5) patients in dataset of CGGA (G). [file 12885_2022_10492_MOESM1_ESM.pdf]
