# Supplementary figures and images for: The prognostic significance of tumor-associated neutrophils and circulating neutrophils in glioblastoma (WHO CNS5 classification)
Source: BMC Cancer. 2023 Jan 6;23:20. doi: 10.1186/s12885-022-10492-9 (PMC9817270; doi:10.1186/s12885-022-10492-9)

A

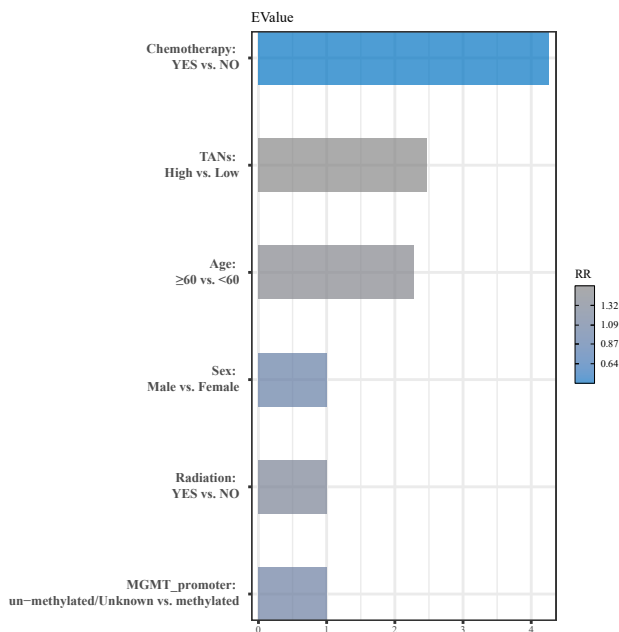

B

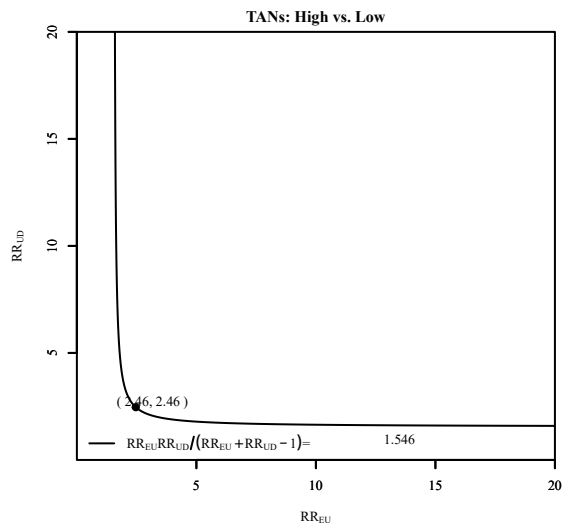

C

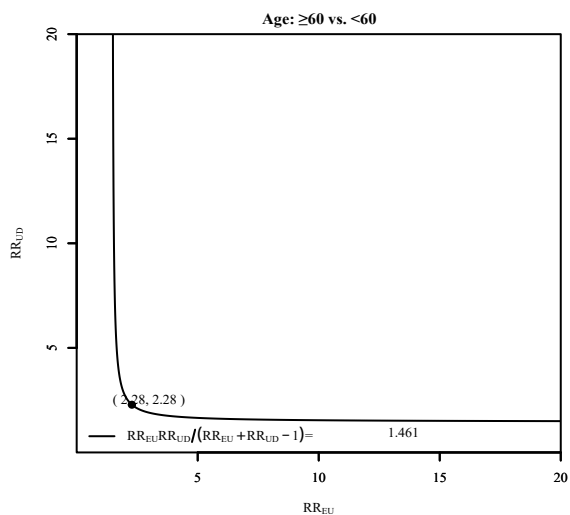

D

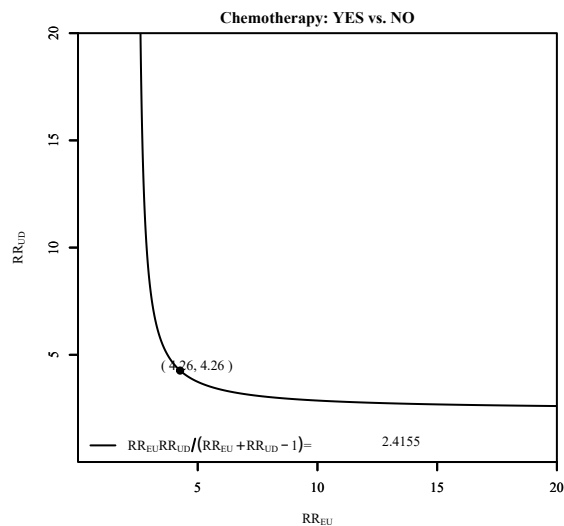

Supplement: Supplementary file 2 — Additional file 2: Fig. S2. Sensitivity analyses in the CGGA cohort. [file 12885_2022_10492_MOESM2_ESM.pdf]
