# Supplementary material for: The prognostic significance of tumor-associated neutrophils and circulating neutrophils in glioblastoma (WHO CNS5 classification)
Source: BMC Cancer. 2023 Jan 6;23:20. doi: 10.1186/s12885-022-10492-9 (PMC9817270; doi:10.1186/s12885-022-10492-9)
Supplement: Supplementary file 3 — Additional file 3: Table S1. Correlation analysis of TANs levels with GSVA scores of hallmark gene sets in dataset of TCGA and CGGA, respectively. [file 12885_2022_10492_MOESM3_ESM.docx]

**Table S1**. Correlation analysis of TANs levels with GSVA scores of hallmark gene sets in dataset of TCGA and CGGA, respectively.

| **TCGA database** | | | |
| --- | --- | --- | --- |
| var | Apoptotic-related genes | Correlation coefficient | p.value |
| TANs | HALLMARK_TNFA_SIGNALING_VIA_NFKB | 0.343414343 | 2.47E-05 |
| TANs | HALLMARK_HYPOXIA | 0.441469928 | 3.45E-08 |
| TANs | HALLMARK_IL6_JAK_STAT3_SIGNALING | 0.380629115 | 2.57E-06 |
| TANs | HALLMARK_COMPLEMENT | 0.361996665 | 8.26E-06 |
| TANs | HALLMARK_MTORC1_SIGNALING | 0.31712377 | 1.04E-04 |
| TANs | HALLMARK_INFLAMMATORY_RESPONSE | 0.371154792 | 4.70E-06 |
| TANs | HALLMARK_GLYCOLYSIS | 0.382136832 | 2.34E-06 |
| TANs | HALLMARK_P53_PATHWAY | 0.306793402 | 1.78E-04 |
| TANs | HALLMARK_ANGIOGENESIS | 0.363990244 | 7.31E-06 |
| TANs | HALLMARK_COAGULATION | 0.32783203 | 5.89E-05 |
| TANs | HALLMARK_IL2_STAT5_SIGNALING | 0.31035254 | 1.48E-04 |
| TANs | HALLMARK_ALLOGRAFT_REJECTION | 0.312199589 | 1.35E-04 |
| TANs | HALLMARK_KRAS_SIGNALING_UP | 0.38586178 | 1.83E-06 |
| **CGGA database** | | | |
| var | Apoptotic-related genes | Correlation coefficient | p.value |
| TANs | HALLMARK_TNFA_SIGNALING_VIA_NFKB | 0.58732483 | 2.01E-17 |
| TANs | HALLMARK_HYPOXIA | 0.537808907 | 2.35E-14 |
| TANs | HALLMARK_CHOLESTEROL_HOMEOSTASIS | 0.200583832 | 8.14E-03 |
| TANs | HALLMARK_MITOTIC_SPINDLE | 0.182443704 | 1.63E-02 |
| TANs | HALLMARK_TGF_BETA_SIGNALING | 0.532452885 | 4.71E-14 |
| TANs | HALLMARK_IL6_JAK_STAT3_SIGNALING | 0.638399734 | 3.45E-21 |
| TANs | HALLMARK_DNA_REPAIR | -0.158335801 | 3.75E-02 |
| TANs | HALLMARK_APOPTOSIS | 0.585409478 | 2.70E-17 |
| TANs | HALLMARK_NOTCH_SIGNALING | 0.238882755 | 1.55E-03 |
| TANs | HALLMARK_ADIPOGENESIS | 0.204934584 | 6.84E-03 |
| TANs | HALLMARK_ESTROGEN_RESPONSE_EARLY | 0.53377738 | 3.97E-14 |
| TANs | HALLMARK_ESTROGEN_RESPONSE_LATE | 0.450353915 | 5.07E-10 |
| TANs | HALLMARK_ANDROGEN_RESPONSE | 0.527606463 | 8.75E-14 |
| TANs | HALLMARK_PROTEIN_SECRETION | 0.469089544 | 7.53E-11 |
| TANs | HALLMARK_INTERFERON_ALPHA_RESPONSE | 0.456637432 | 2.71E-10 |
| TANs | HALLMARK_INTERFERON_GAMMA_RESPONSE | 0.584772188 | 2.98E-17 |
| TANs | HALLMARK_APICAL_JUNCTION | 0.431642663 | 3.04E-09 |
| TANs | HALLMARK_APICAL_SURFACE | 0.374433988 | 3.87E-07 |
| TANs | HALLMARK_HEDGEHOG_SIGNALING | 0.21783013 | 3.99E-03 |
| TANs | HALLMARK_COMPLEMENT | 0.623137277 | 5.44E-20 |
| TANs | HALLMARK_UNFOLDED_PROTEIN_RESPONSE | 0.359219125 | 1.21E-06 |
| TANs | HALLMARK_PI3K_AKT_MTOR_SIGNALING | 0.523045603 | 1.55E-13 |
| TANs | HALLMARK_MTORC1_SIGNALING | 0.414173488 | 1.47E-08 |
| TANs | HALLMARK_EPITHELIAL_MESENCHYMAL_TRANSITION | 0.53645423 | 2.80E-14 |
| TANs | HALLMARK_INFLAMMATORY_RESPONSE | 0.662188411 | 3.39E-23 |
| TANs | HALLMARK_XENOBIOTIC_METABOLISM | 0.277584482 | 2.18E-04 |
| TANs | HALLMARK_OXIDATIVE_PHOSPHORYLATION | -0.212511254 | 5.00E-03 |
| TANs | HALLMARK_GLYCOLYSIS | 0.393590995 | 8.45E-08 |
| TANs | HALLMARK_REACTIVE_OXYGEN_SPECIES_PATHWAY | 0.166627536 | 2.84E-02 |
| TANs | HALLMARK_P53_PATHWAY | 0.411237775 | 1.90E-08 |
| TANs | HALLMARK_UV_RESPONSE_UP | 0.330820833 | 8.77E-06 |
| TANs | HALLMARK_UV_RESPONSE_DN | 0.512460088 | 5.70E-13 |
| TANs | HALLMARK_ANGIOGENESIS | 0.554576947 | 2.44E-15 |
| TANs | HALLMARK_HEME_METABOLISM | 0.454579298 | 3.33E-10 |
| TANs | HALLMARK_COAGULATION | 0.445528388 | 8.13E-10 |
| TANs | HALLMARK_IL2_STAT5_SIGNALING | 0.630401687 | 1.49E-20 |
| TANs | HALLMARK_BILE_ACID_METABOLISM | 0.212295342 | 5.05E-03 |
| TANs | HALLMARK_ALLOGRAFT_REJECTION | 0.576694044 | 1.01E-16 |
| TANs | HALLMARK_KRAS_SIGNALING_UP | 0.611040376 | 4.36E-19 |
