# Supplementary material for: The prognostic significance of tumor-associated neutrophils and circulating neutrophils in glioblastoma (WHO CNS5 classification)
Source: BMC Cancer. 2023 Jan 6;23:20. doi: 10.1186/s12885-022-10492-9 (PMC9817270; doi:10.1186/s12885-022-10492-9)
Supplement: Supplementary file 4 — Additional file 4: Table S2. Correlation analysis of TANs levels with GSVA scores of KEGG pathways in dataset of TCGA and CGGA, respectively. [file 12885_2022_10492_MOESM4_ESM.docx]

**Table S2**. Correlation analysis of TANs levels with GSVA scores of KEGG pathways in dataset of TCGA and CGGA, respectively.

| **TCGA database** | | | |
| --- | --- | --- | --- |
| var | Apoptotic-related genes | Correlation coefficient | p.value |
| TANs | KEGG_OTHER_GLYCAN_DEGRADATION | 0.309774132 | 1.53E-04 |
| TANs | KEGG_LYSOSOME | 0.345731831 | 2.16E-05 |
| TANs | KEGG_PATHOGENIC_ESCHERICHIA_COLI_INFECTION | 0.360091774 | 9.26E-06 |
| TANs | KEGG_SYSTEMIC_LUPUS_ERYTHEMATOSUS | 0.328842316 | 5.57E-05 |
| TANs | KEGG_GLYCOLYSIS_GLUCONEOGENESIS | 0.339442607 | 3.09E-05 |
| TANs | KEGG_PENTOSE_PHOSPHATE_PATHWAY | 0.310784418 | 1.45E-04 |
| TANs | KEGG_FRUCTOSE_AND_MANNOSE_METABOLISM | 0.374143233 | 3.89E-06 |
| TANs | KEGG_GALACTOSE_METABOLISM | 0.458008541 | 9.15E-09 |
| TANs | KEGG_STARCH_AND_SUCROSE_METABOLISM | 0.320686763 | 8.64E-05 |
| TANs | KEGG_AMINO_SUGAR_AND_NUCLEOTIDE_SUGAR_METABOLISM | 0.372623948 | 4.28E-06 |
| TANs | KEGG_CYTOKINE_CYTOKINE_RECEPTOR_INTERACTION | 0.319148198 | 9.37E-05 |
| TANs | KEGG_CHEMOKINE_SIGNALING_PATHWAY | 0.326675214 | 6.27E-05 |
| TANs | KEGG_APOPTOSIS | 0.431309227 | 7.52E-08 |
| TANs | KEGG_COMPLEMENT_AND_COAGULATION_CASCADES | 0.314925819 | 1.17E-04 |
| TANs | KEGG_TOLL_LIKE_RECEPTOR_SIGNALING_PATHWAY | 0.364649629 | 7.02E-06 |
| TANs | KEGG_NOD_LIKE_RECEPTOR_SIGNALING_PATHWAY | 0.317374413 | 1.03E-04 |
| TANs | KEGG_HEMATOPOIETIC_CELL_LINEAGE | 0.326698351 | 6.26E-05 |
| TANs | KEGG_NATURAL_KILLER_CELL_MEDIATED_CYTOTOXICITY | 0.34812644 | 1.88E-05 |
| TANs | KEGG_B_CELL_RECEPTOR_SIGNALING_PATHWAY | 0.326139223 | 6.45E-05 |
| TANs | KEGG_LEUKOCYTE_TRANSENDOTHELIAL_MIGRATION | 0.368004396 | 5.71E-06 |
| TANs | KEGG_EPITHELIAL_CELL_SIGNALING_IN_HELICOBACTER_PYLORI_INFECTION | 0.39902249 | 7.61E-07 |
| TANs | KEGG_LEISHMANIA_INFECTION | 0.347320525 | 1.97E-05 |
| TANs | KEGG_RENAL_CELL_CARCINOMA | 0.321805019 | 8.14E-05 |
| TANs | KEGG_PANCREATIC_CANCER | 0.33403642 | 4.19E-05 |
| **CGGA database** | | | |
| var | Apoptotic-related genes | Correlation coefficient | p.value |
| TANs | KEGG_N_GLYCAN_BIOSYNTHESIS | 0.433739103 | 2.50E-09 |
| TANs | KEGG_OTHER_GLYCAN_DEGRADATION | 0.386178 | 1.54E-07 |
| TANs | KEGG_O_GLYCAN_BIOSYNTHESIS | 0.496977076 | 3.53E-12 |
| TANs | KEGG_GLYCOSAMINOGLYCAN_DEGRADATION | 0.359610321 | 1.18E-06 |
| TANs | KEGG_GLYCOSAMINOGLYCAN_BIOSYNTHESIS_KERATAN_SULFATE | 0.367631585 | 6.50E-07 |
| TANs | KEGG_SPHINGOLIPID_METABOLISM | 0.480658273 | 2.19E-11 |
| TANs | KEGG_GLYCOSPHINGOLIPID_BIOSYNTHESIS_GLOBO_SERIES | 0.346401354 | 3.03E-06 |
| TANs | KEGG_GLYCOSPHINGOLIPID_BIOSYNTHESIS_GANGLIO_SERIES | 0.327652956 | 1.08E-05 |
| TANs | KEGG_RIBOFLAVIN_METABOLISM | 0.345261429 | 3.28E-06 |
| TANs | KEGG_NICOTINATE_AND_NICOTINAMIDE_METABOLISM | 0.357743723 | 1.35E-06 |
| TANs | KEGG_PANTOTHENATE_AND_COA_BIOSYNTHESIS | 0.421521476 | 7.67E-09 |
| TANs | KEGG_SNARE_INTERACTIONS_IN_VESICULAR_TRANSPORT | 0.316318713 | 2.24E-05 |
| TANs | KEGG_LYSOSOME | 0.451911735 | 4.35E-10 |
| TANs | KEGG_RENIN_ANGIOTENSIN_SYSTEM | 0.415777741 | 1.28E-08 |
| TANs | KEGG_PATHOGENIC_ESCHERICHIA_COLI_INFECTION | 0.47987588 | 2.38E-11 |
| TANs | KEGG_SYSTEMIC_LUPUS_ERYTHEMATOSUS | 0.442521865 | 1.09E-09 |
| TANs | KEGG_PRIMARY_IMMUNODEFICIENCY | 0.359900526 | 1.15E-06 |
| TANs | KEGG_HYPERTROPHIC_CARDIOMYOPATHY_HCM | 0.438310411 | 1.63E-09 |
| TANs | KEGG_ARRHYTHMOGENIC_RIGHT_VENTRICULAR_CARDIOMYOPATHY_ARVC | 0.390332574 | 1.10E-07 |
| TANs | KEGG_DILATED_CARDIOMYOPATHY | 0.393034962 | 8.85E-08 |
| TANs | KEGG_GALACTOSE_METABOLISM | 0.385959766 | 1.57E-07 |
| TANs | KEGG_STARCH_AND_SUCROSE_METABOLISM | 0.519394825 | 2.45E-13 |
| TANs | KEGG_AMINO_SUGAR_AND_NUCLEOTIDE_SUGAR_METABOLISM | 0.398933087 | 5.43E-08 |
| TANs | KEGG_PPAR_SIGNALING_PATHWAY | 0.345261429 | 3.28E-06 |
| TANs | KEGG_MAPK_SIGNALING_PATHWAY | 0.493818485 | 5.06E-12 |
| TANs | KEGG_CYTOKINE_CYTOKINE_RECEPTOR_INTERACTION | 0.57242571 | 1.91E-16 |
| TANs | KEGG_CHEMOKINE_SIGNALING_PATHWAY | 0.635313115 | 6.10E-21 |
| TANs | KEGG_P53_SIGNALING_PATHWAY | 0.331309538 | 8.49E-06 |
| TANs | KEGG_ENDOCYTOSIS | 0.403139897 | 3.81E-08 |
| TANs | KEGG_MTOR_SIGNALING_PATHWAY | 0.375688834 | 3.52E-07 |
| TANs | KEGG_APOPTOSIS | 0.637646362 | 3.97E-21 |
| TANs | KEGG_VASCULAR_SMOOTH_MUSCLE_CONTRACTION | 0.390870033 | 1.06E-07 |
| TANs | KEGG_DORSO_VENTRAL_AXIS_FORMATION | 0.420112241 | 8.71E-09 |
| TANs | KEGG_TGF_BETA_SIGNALING_PATHWAY | 0.477837481 | 2.97E-11 |
| TANs | KEGG_VEGF_SIGNALING_PATHWAY | 0.481664703 | 1.96E-11 |
| TANs | KEGG_FOCAL_ADHESION | 0.542304761 | 1.30E-14 |
| TANs | KEGG_ECM_RECEPTOR_INTERACTION | 0.414651746 | 1.41E-08 |
| TANs | KEGG_CELL_ADHESION_MOLECULES_CAMS | 0.542810879 | 1.21E-14 |
| TANs | KEGG_ADHERENS_JUNCTION | 0.478256537 | 2.84E-11 |
| TANs | KEGG_TIGHT_JUNCTION | 0.353226974 | 1.87E-06 |
| TANs | KEGG_COMPLEMENT_AND_COAGULATION_CASCADES | 0.462763077 | 1.45E-10 |
| TANs | KEGG_ANTIGEN_PROCESSING_AND_PRESENTATION | 0.533313052 | 4.22E-14 |
| TANs | KEGG_TOLL_LIKE_RECEPTOR_SIGNALING_PATHWAY | 0.652240187 | 2.46E-22 |
| TANs | KEGG_NOD_LIKE_RECEPTOR_SIGNALING_PATHWAY | 0.633769224 | 8.09E-21 |
| TANs | KEGG_RIG_I_LIKE_RECEPTOR_SIGNALING_PATHWAY | 0.472931857 | 5.02E-11 |
| TANs | KEGG_CYTOSOLIC_DNA_SENSING_PATHWAY | 0.411846045 | 1.81E-08 |
| TANs | KEGG_JAK_STAT_SIGNALING_PATHWAY | 0.657575314 | 8.59E-23 |
| TANs | KEGG_HEMATOPOIETIC_CELL_LINEAGE | 0.516872364 | 3.34E-13 |
| TANs | KEGG_NATURAL_KILLER_CELL_MEDIATED_CYTOTOXICITY | 0.65363201 | 1.88E-22 |
| TANs | KEGG_T_CELL_RECEPTOR_SIGNALING_PATHWAY | 0.617925197 | 1.35E-19 |
| TANs | KEGG_B_CELL_RECEPTOR_SIGNALING_PATHWAY | 0.624356138 | 4.39E-20 |
| TANs | KEGG_FC_EPSILON_RI_SIGNALING_PATHWAY | 0.529704064 | 6.70E-14 |
| TANs | KEGG_FC_GAMMA_R_MEDIATED_PHAGOCYTOSIS | 0.530892743 | 5.76E-14 |
| TANs | KEGG_LEUKOCYTE_TRANSENDOTHELIAL_MIGRATION | 0.611689274 | 3.90E-19 |
| TANs | KEGG_INTESTINAL_IMMUNE_NETWORK_FOR_IGA_PRODUCTION | 0.443631608 | 9.77E-10 |
| TANs | KEGG_NEUROTROPHIN_SIGNALING_PATHWAY | 0.442589192 | 1.08E-09 |
| TANs | KEGG_REGULATION_OF_ACTIN_CYTOSKELETON | 0.554575786 | 2.45E-15 |
| TANs | KEGG_INSULIN_SIGNALING_PATHWAY | 0.371057163 | 5.02E-07 |
| TANs | KEGG_PROGESTERONE_MEDIATED_OOCYTE_MATURATION | 0.339600112 | 4.86E-06 |
| TANs | KEGG_ADIPOCYTOKINE_SIGNALING_PATHWAY | 0.499703841 | 2.58E-12 |
| TANs | KEGG_TYPE_II_DIABETES_MELLITUS | 0.413290104 | 1.59E-08 |
| TANs | KEGG_TYPE_I_DIABETES_MELLITUS | 0.45926901 | 2.08E-10 |
| TANs | KEGG_ALDOSTERONE_REGULATED_SODIUM_REABSORPTION | 0.357763457 | 1.35E-06 |
| TANs | KEGG_PRION_DISEASES | 0.478445751 | 2.78E-11 |
| TANs | KEGG_EPITHELIAL_CELL_SIGNALING_IN_HELICOBACTER_PYLORI_INFECTION | 0.483265474 | 1.65E-11 |
| TANs | KEGG_LEISHMANIA_INFECTION | 0.612704991 | 3.29E-19 |
| TANs | KEGG_PATHWAYS_IN_CANCER | 0.563590711 | 6.87E-16 |
| TANs | KEGG_COLORECTAL_CANCER | 0.454068538 | 3.51E-10 |
| TANs | KEGG_RENAL_CELL_CARCINOMA | 0.454755743 | 3.27E-10 |
| TANs | KEGG_PANCREATIC_CANCER | 0.567463206 | 3.94E-16 |
| TANs | KEGG_ENDOMETRIAL_CANCER | 0.368396565 | 6.14E-07 |
| TANs | KEGG_GLIOMA | 0.308588815 | 3.62E-05 |
| TANs | KEGG_PROSTATE_CANCER | 0.493411038 | 5.30E-12 |
| TANs | KEGG_THYROID_CANCER | 0.448190147 | 6.27E-10 |
| TANs | KEGG_MELANOMA | 0.413802026 | 1.52E-08 |
| TANs | KEGG_BLADDER_CANCER | 0.380288001 | 2.46E-07 |
| TANs | KEGG_CHRONIC_MYELOID_LEUKEMIA | 0.455731992 | 2.97E-10 |
| TANs | KEGG_ACUTE_MYELOID_LEUKEMIA | 0.521440189 | 1.90E-13 |
| TANs | KEGG_SMALL_CELL_LUNG_CANCER | 0.551840895 | 3.57E-15 |
| TANs | KEGG_NON_SMALL_CELL_LUNG_CANCER | 0.402344736 | 4.08E-08 |
| TANs | KEGG_ASTHMA | 0.411930784 | 1.79E-08 |
| TANs | KEGG_AUTOIMMUNE_THYROID_DISEASE | 0.44040453 | 1.33E-09 |
| TANs | KEGG_ALLOGRAFT_REJECTION | 0.485876157 | 1.23E-11 |
| TANs | KEGG_GRAFT_VERSUS_HOST_DISEASE | 0.479524152 | 2.48E-11 |
| TANs | KEGG_VIRAL_MYOCARDITIS | 0.520554484 | 2.12E-13 |
