# Supplementary material for: The prognostic significance of tumor-associated neutrophils and circulating neutrophils in glioblastoma (WHO CNS5 classification)
Source: BMC Cancer. 2023 Jan 6;23:20. doi: 10.1186/s12885-022-10492-9 (PMC9817270; doi:10.1186/s12885-022-10492-9)
Supplement: Supplementary file 5 — Additional file 5: Table S3. Correlation analysis of TANs levels with apoptotic-related genes in dataset of TCGA and CGGA, respectively. [file 12885_2022_10492_MOESM5_ESM.docx]

**Table S3**. Correlation analysis of TANs levels with apoptotic-related genes in dataset of TCGA and CGGA, respectively.

| **TCGA database** | | | |
| --- | --- | --- | --- |
| var | Apoptotic-related genes | Correlation coefficient | p.value |
| TANs | BIRC3 | 0.350170486 | 1.47E-05 |
| TANs | CASP10 | 0.330161087 | 5.18E-05 |
| TANs | CSF2RB | 0.396662586 | 8.93E-07 |
| TANs | FAS | 0.335490153 | 3.86E-05 |
| TANs | IL1A | 0.358024929 | 1.05E-05 |
| TANs | IL1B | 0.368671493 | 5.48E-06 |
| TANs | MYD88 | 0.347420782 | 1.96E-05 |
| TANs | PIK3CD | 0.310067192 | 1.50E-04 |
| TANs | PIK3CG | 0.352102031 | 1.49E-05 |
| TANs | TNFRSF10C | 0.459608803 | 8.01E-09 |
| TANs | TNFRSF10D | 0.39740757 | 6.81E-07 |
| **CGGA database** | | | |
| var | Apoptotic-related genes | Correlation coefficient | p.value |
| TANs | BIRC3 | 0.444697358 | 8.82E-10 |
| TANs | CASP10 | 0.414930017 | 1.38E-08 |
| TANs | CSF2RB | 0.640297736 | 2.42E-21 |
| TANs | FAS | 0.484801426 | 1.39E-11 |
| TANs | IL1A | 0.369407289 | 5.68E-07 |
| TANs | IL1B | 0.474834535 | 4.10E-11 |
| TANs | MYD88 | 0.491137265 | 6.86E-12 |
| TANs | PIK3CD | 0.311877368 | 2.95E-05 |
| TANs | PIK3CG | 0.559594237 | 1.21E-15 |
| TANs | TNFRSF10C | 0.461497185 | 1.65E-10 |
| TANs | TNFRSF10D | 0.425544596 | 5.33E-09 |
