# Supplementary material for: The prognostic significance of tumor-associated neutrophils and circulating neutrophils in glioblastoma (WHO CNS5 classification)
Source: BMC Cancer. 2023 Jan 6;23:20. doi: 10.1186/s12885-022-10492-9 (PMC9817270; doi:10.1186/s12885-022-10492-9)
Supplement: Supplementary file 6 — Additional file 6: Table S4. Correlation analysis of TANs levels with neutrophils function-related marker genes in dataset of TCGA and CGGA, respectively. [file 12885_2022_10492_MOESM6_ESM.docx]

**Table S4**. Correlation analysis of TANs levels with neutrophils function-related marker genes in dataset of TCGA and CGGA, respectively.

| **TCGA database** | | | |
| --- | --- | --- | --- |
| var | Neutrophils function-related marker genes | Correlation coefficient | p.value |
| TANs | CXCR4 | 0.29556843 | 3.10E-04 |
| TANs | TGFBR1 | 0.212458909 | 1.02E-02 |
| TANs | CXCR1 | 0.700241394 | 7.91E-23 |
| TANs | CD86 | 0.291912892 | 3.70E-04 |
| TANs | PILRA | 0.301564594 | 2.31E-04 |
| TANs | LILRB2 | 0.421491714 | 1.56E-07 |
| TANs | CD200R1 | 0.305506364 | 1.77E-04 |
| TANs | TNFSF10 | 0.274992529 | 8.15E-04 |
| TANs | S100A9 | 0.627859987 | 0.00E+00 |
| TANs | S100A8 | 0.591829023 | 0.00E+00 |
| TANs | PROK2 | 0.452406384 | 9.88E-09 |
| TANs | MMP9 | 0.390909354 | 1.31E-06 |
| TANs | PDGFB | 0.282781757 | 5.70E-04 |
| TANs | ARG1 | 0.368455757 | 4.74E-06 |
| **CGGA database** | | | |
| var | Neutrophils function-related marker genes | Correlation coefficient | p.value |
| TANs | CXCR4 | 0.419321966 | 9.35E-09 |
| TANs | TGFBR1 | 0.411303392 | 1.89E-08 |
| TANs | CXCR1 | 0.569350042 | 2.99E-16 |
| TANs | CD86 | 0.486045005 | 1.21E-11 |
| TANs | PILRA | 0.271582562 | 3.01E-04 |
| TANs | LILRB2 | 0.436114579 | 2.00E-09 |
| TANs | CD200R1 | 0.431987186 | 2.95E-09 |
| TANs | TNFSF10 | 0.448812245 | 5.90E-10 |
| TANs | S100A9 | 0.542498715 | 1.26E-14 |
| TANs | S100A8 | 0.394331598 | 7.95E-08 |
| TANs | PROK2 | 0.327385608 | 1.10E-05 |
| TANs | MMP9 | 0.195414801 | 9.98E-03 |
| TANs | AGTR1 | 0.208343612 | 5.95E-03 |
| TANs | IFNAR1 | 0.405083233 | 3.23E-08 |
| TANs | PDGFB | 0.163200581 | 3.19E-02 |
| TANs | ARG1 | 0.268779401 | 3.49E-04 |
