# Supplementary material for: The prognostic significance of tumor-associated neutrophils and circulating neutrophils in glioblastoma (WHO CNS5 classification)
Source: BMC Cancer. 2023 Jan 6;23:20. doi: 10.1186/s12885-022-10492-9 (PMC9817270; doi:10.1186/s12885-022-10492-9)
Supplement: Supplementary file 7 — Additional file 7: Table S5. Characteristics of the study population based on the level of peripheral blood neutrophils before radiotherapy. [file 12885_2022_10492_MOESM7_ESM.docx]

**Table S5.** Characteristics of the study population based on the level of peripheral blood neutrophils before radiotherapy.

| Variables | Total  (n = 143) | High  (n = 113) | Low  (n = 30) | p |
| --- | --- | --- | --- | --- |
| Age |  |  |  | 0.996 |
| <60 | 93 (65%) | 74 (65%) | 19 (63%) |  |
| ≥60 | 50 (35%) | 39 (35%) | 11 (37%) |  |
| Sex |  |  |  | 0.23 |
| Female | 51 (36%) | 37 (33%) | 14 (47%) |  |
| Male | 92 (64%) | 76 (67%) | 16 (53%) |  |
| MGMT promoter |  |  |  | 0.987 |
| Methylated | 55 (38%) | 44 (39%) | 11 (37%) |  |
| Un-methylated/Unknown | 88 (62%) | 69 (61%) | 19 (63%) |  |
