# Supplementary material for: The prognostic significance of tumor-associated neutrophils and circulating neutrophils in glioblastoma (WHO CNS5 classification)
Source: BMC Cancer. 2023 Jan 6;23:20. doi: 10.1186/s12885-022-10492-9 (PMC9817270; doi:10.1186/s12885-022-10492-9)

Supplementary File 1

The distribution of the TANs levels between molGBM and histoGBM .

|  | molGBM | histoGBM | Z | P |
| --- | --- | --- | --- | --- |
| TANs  Median(IQR) | 0.014(0.009 - 0.02) | 0.028(0.019 - 0.042) | 780 | ＜0.001 |


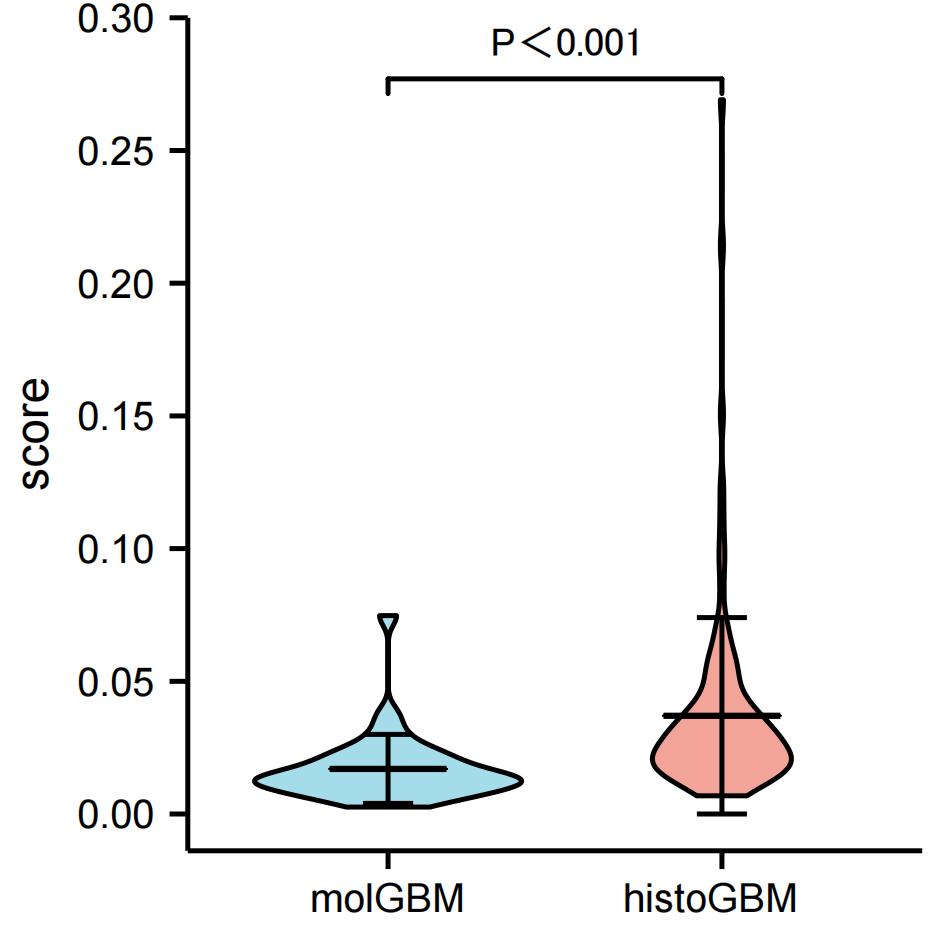

Supplement: Supplementary file 8 — Additional file 8: Supplementary File 1. The distribution of the TANs levels between molGBM and histoGBM. [file 12885_2022_10492_MOESM8_ESM.docx]
